# CRP at diagnosis in psoriatic arthritis: what it means and associations with long-term outcomes

**DOI:** 10.1093/rheumatology/keag398

**Published:** 2026-08-07

**Authors:** Angeliki E Dimopoulou, Charalampos Papagoras, Niki Kyriazi, Sousana Gazi, Evangelia Mole, Michael Krikelis, Paraskevi V Voulgari, Evripidis Kaltsonoudis, Nikolaos Koletsos, Pelagia Katsimpri, Dimitrios Boumpas, Dimitrios Katsifis-Nezis, Nikolaos Kougkas, Theodoros Dimitroulas, Petros P Sfikakis, Maria G Tektonidou, Konstantinos D Vassilakis, Dimitrios Bogdanos, Theodora Simopoulou, Christos Koutsianas, Eugenia Mavrea, Gkikas Katsifis, Konstantinos Kottas, Maria Konsta, Matthoula Tziafalia, Evangelia Kataxaki, Eleni Kalavri, Kalliopi Klavdianou, Anastasios Karamanakos, Ioannis Xynogalas, Dimitrios Daoussis, George Iliopoulos, Ilias Bournazos, Konstantinos Georganas, Dimos Patrikos, Dimitrios Vassilopoulos, George E Fragoulis

**Affiliations:** Joint Academic Rheumatology Program, First Department of Propedeutic and Internal Medicine, National and Kapodistrian University of Athens, School of Medicine, Athens, Greece; First Department of Internal Medicine, University Hospital of Alexandroupolis, Democritus University of Thrace, Alexandroupolis, Greece; Department of Rheumatology, Evangelismos Athens General Hospital, Athens, Greece; Department of Rheumatology, KAT Hospital, Athens, Greece; Department of Rheumatology, KAT Hospital, Athens, Greece; Department of Rheumatology, KAT Hospital, Athens, Greece; Department of Rheumatology, School of Health Sciences, Faculty of Medicine, University of Ioannina, Ioannina, Greece; Department of Rheumatology, School of Health Sciences, Faculty of Medicine, University of Ioannina, Ioannina, Greece; Department of Rheumatology, School of Health Sciences, Faculty of Medicine, University of Ioannina, Ioannina, Greece; Department of Rheumatology, KAT Hospital, Athens, Greece; Joint Academic Rheumatology Program, 4th Department of Internal Medicine, Attikon University Hospital, National and Kapodistrian University of Athens, School of Medicine, Athens, Greece; Joint Academic Rheumatology Program, 4th Department of Internal Medicine, Attikon University Hospital, National and Kapodistrian University of Athens, School of Medicine, Athens, Greece; 4th Department of Medicine, Aristotle University, Thessaloniki, Greece; 4th Department of Medicine, Aristotle University, Thessaloniki, Greece; Joint Academic Rheumatology Program, First Department of Propedeutic and Internal Medicine, National and Kapodistrian University of Athens, School of Medicine, Athens, Greece; Joint Academic Rheumatology Program, First Department of Propedeutic and Internal Medicine, National and Kapodistrian University of Athens, School of Medicine, Athens, Greece; Joint Academic Rheumatology Program, First Department of Propedeutic and Internal Medicine, National and Kapodistrian University of Athens, School of Medicine, Athens, Greece; Department of Rheumatology and Clinical Immunology, University of Thessaly, Larissa, Greece; Department of Rheumatology and Clinical Immunology, University of Thessaly, Larissa, Greece; Joint Academic Rheumatology Program, Clinical Immunology–Rheumatology Unit, 2nd Department of Medicine and Laboratory, National and Kapodistrian University of Athens, School of Medicine, General Hospital of Athens “Hippokration”, Athens, Greece; Joint Academic Rheumatology Program, Clinical Immunology–Rheumatology Unit, 2nd Department of Medicine and Laboratory, National and Kapodistrian University of Athens, School of Medicine, General Hospital of Athens “Hippokration”, Athens, Greece; Rheumatology Clinic, Naval Hospital of Athens, Athens, Greece; Rheumatology Clinic, Naval Hospital of Athens, Athens, Greece; Rheumatology Unit, Sismanoglio Hospital, Athens, Greece; Rheumatology Unit, Sismanoglio Hospital, Athens, Greece; Rheumatology Department, General Hospital Elefsinas Thriaseio, Athens, Greece; Department of Rheumatology, Asklepieion General Hospital, Athens, Greece; Department of Rheumatology, Asklepieion General Hospital, Athens, Greece; Department of Rheumatology, Evangelismos Athens General Hospital, Athens, Greece; Department of Rheumatology, Evangelismos Athens General Hospital, Athens, Greece; Department of Rheumatology, Patras University Hospital, University of Patras Medical School, Patras, Greece; Private Practice, Athens, Greece; Private Practice, Athens, Greece; Private Practice, Athens, Greece; Private Practice, Athens, Greece; Joint Academic Rheumatology Program, Clinical Immunology–Rheumatology Unit, 2nd Department of Medicine and Laboratory, National and Kapodistrian University of Athens, School of Medicine, General Hospital of Athens “Hippokration”, Athens, Greece; Joint Academic Rheumatology Program, First Department of Propedeutic and Internal Medicine, National and Kapodistrian University of Athens, School of Medicine, Athens, Greece; School of Infection and Immunity, University of Glasgow, Glasgow, UK

**Keywords:** CRP, psoriatic arthritis, long-term outcomes

## Abstract

**Objective:**

The role of CRP in PsA as a diagnostic and prognostic marker is debated. We compared clinical and epidemiological features, as well as long-term outcomes, between patients with ‘elevated’ (>0.5 mg/dl) and patients with ‘normal’ (≤0.5 mg/dl) CRP at diagnosis.

**Methods:**

In this real-world study, we analysed data from 609 PsA patients categorized by their CRP at diagnosis. Demographics, clinical characteristics, comorbidities, and long-term outcomes were compared. The statistically significant variables in the univariate analyses were used to build two multivariable logistic regression models: (i) assessing associations between CRP and features at PsA diagnosis and (ii) examining its link with long-term outcomes (including ‘difficult to manage’ and ‘persistent disease’) and characteristics throughout the disease course.

**Results:**

Of 609 patients, 132 (21.7%) displayed normal and 477 (78.3%) elevated CRP values at diagnosis. By univariate analysis, elevated CRP was associated with longer disease follow-up, BMI of >25 kg/m^2^, enthesitis (at diagnosis and during the disease course), hypertension, hyperuricaemia, new bone formation, and ‘persistent disease’. By multivariable analyses, elevated CRP at diagnosis was independently linked with BMI of >25 kg/m^2^ [odds ratio (OR) 1.69, 95% CI 1.05–2.72], enthesitis (OR 2.04, 95% CI 1.24–3.38), hypertension (OR 2.07, 95% CI 1.04–4.11), new bone formation (OR 2.57, 95% CI 1.33–4.94) and ‘persistent disease’ (OR 4.36, 95% CI 2.22–8.59).

**Conclusion:**

Overall PsA characteristics are comparable, irrespective of the CRP levels at diagnosis, apart from new bone formation, enthesitis, ‘persistent disease’ and increased cardiometabolic burden, which were independently associated with elevated CRP levels at PsA diagnosis.

Rheumatology key messages

- CRP has limited value for monitoring PsA activity but may help identify distinct disease phenotypes and predict long-term outcomes.
- Elevated CRP at PsA diagnosis is associated with a persistent disease phenotype, enthesitis, new bone formation, and increased cardiometabolic burden.

## Introduction

PsA is a complex, chronic, immune-mediated inflammatory disease involving multiple organ systems. Its multifaceted presentation consists of both musculoskeletal manifestations, such as peripheral arthritis, axial disease, dactylitis and enthesitis, and extra-musculoskeletal manifestations, including psoriasis, nail involvement, IBD, and eye involvement, most commonly uveitis [1, 2]. Additionally, a variety of associated comorbidities, such as cardiovascular disease, metabolic syndrome, mental health disorders, and FM, further augment PsA’s heterogeneity and burden [3–5]. Both genetic and environmental (especially intestinal microbiome and mechanical stress) factors have been implicated in the disease’s pathogenesis, with key roles being attributed to the IL-23/IL-17 pathway, TNF and IL-22 [1, 6, 7].

CRP is a pentraxin produced by hepatocytes in response to inflammatory proteins, such as IL-1β, IL-6 and TNF, that aids in complement binding and macrophage phagocytosis [8–10]. It is one of the most commonly used biomarkers of systemic inflammation and tissue injury, being highly sensitive, readily available, and cost-efficient [9, 11], and it is particularly suitable for recognizing and monitoring acute inflammatory states [10, 11]. However, caution should be exerted in interpretation of this acute-phase reactant’s levels—even though an increase can serve as confirmation of clinical suspicion of inflammation, it is not specific, and normal levels cannot be evidence enough to exclude it [9].

Given that this acute-phase reactant is found to be elevated in only about half of the patients with active PsA [12], a handful of studies have come out investigating its role in disease activity measurement in PsA. Within the PsA community, the most appropriate index for disease activity, evaluation, and treatment tailoring has long been debated, with the potential redundancy of CRP in routine evaluation being a concern [12–14]. The Disease Activity index for PSoriatic Arthritis (DAPSA) and its clinical variant (cDAPSA—which is calculated with the same parameters as the former, omitting CRP)—have been shown to result in similar patient categorization according to disease activity [13, 15, 16]. On the other hand, cDAPSA remission scores and Minimal Disease Activity (MDA) state have been documented as not being aligned with the indications of CRP levels [17]. In addition, in a study examining definitions of remission and low disease activity, proportions of patients with increased CRP were comparable across the definitions, so it was argued that CRP might not provide additional information in terms of monitoring [14].

Nevertheless, there are some data to support that CRP at diagnosis can serve as a predictor for adverse outcomes, including structural damage [11, 18–21]. Therefore, with this real-world nationwide study, we aimed to investigate whether CRP serum levels at diagnosis could be linked to different subgroups of PsA, with potentially different clinical and epidemiological features, and different disease courses.

## Methods

Data was extracted from the Greek Rheumatology Society’s multicentre study [22] of PsA patients (fulfilling the CASPAR criteria). The data for all patients who visited the participating clinics and private practices between 1 January 2022 and 31 December 2022 (time of assessment) were analysed cross-sectionally. The protocol and data collected have been described in detail elsewhere [22]. In summary, in the original cohort, adult individuals (≥18 years) with PsA attending University Hospitals, National Health System Hospitals, or private practices across Greece were enrolled regardless of disease duration (newly diagnosed or established PsA) and irrespective of prior treatment exposure [conventional synthetic (cs)DMARD-treated, biologic (b)DMARD–naïve, bDMARD recipients, or targeted synthetic (ts)DMARD recipients].

Briefly, the following features were recorded: demographics (e.g. age and working status), time of follow-up (time between PsA diagnosis and the time of assessment), clinical and laboratory characteristics (e.g. joint counts, enthesitis, IBD, BSA), indices (e.g. DAPSA, HAQ) and comorbidities at the time of PsA diagnosis and thereafter. Past and present anti-rheumatic treatments, as well as last available (prior to time of assessment) hands and spine (including SI joints) imaging were recorded.

For this project, we considered the cut-off for elevated and normal CRP to be 0.5 mg/dl. Accordingly, patients were categorized into a ‘normal’ (CRP ≤ 0.5 mg/dl) and an ‘elevated’ (CRP > 0.5 mg/dl) CRP group at the time of PsA diagnosis. A dichotomous categorization of patients was proposed, to allow for a more clinically applicable, efficient, and easily interpretable comparison.

Univariate analyses were conducted examining the associations of elevated or normal CRP at diagnosis with demographics, comorbidities, disease characteristics at the time of PsA diagnosis and at any time during the disease course, as well as with long-term outcomes. The latter included radiographic damage (erosions and new bone formation on hand x-rays, sacroiliitis, and syndesmophytes on spine X-rays) and arthroplasties, as well as HAQ, difficult to manage (D2M) disease, and treatment refractory (TR) disease status based on data collected on the time of assessment. As per the recently published EULAR points for consideration [23], D2M was defined as evidence of ‘persistent disease’ [(a) not being in MDA and/or (b) having at least one active extra-musculoskeletal manifestation and/or (c) demonstrating ‘inflammatory activity’ (CRP > 0.5 mg/dl and/or positive findings in MRI in the 6 months prior to the time of assessment)] while having failed to achieve and maintain response with at least two b/tsDMARDs, with at least two mechanisms of action. According to the same definition, TR disease was defined as: having at least two of the ‘persistent disease’ characteristics (with ‘inflammatory activity’ being mandatory) and failure to achieve and maintain response with at least two b/tsDMARDs, with at least two mechanisms of action. Additionally, according to the TR definition, patients with co-morbidities, and psychosocial and other factors that can be considered drivers of ‘persistent disease’ should be excluded. For the purpose of this study, we viewed depression and neoplasia as such drivers.

For the univariate analysis, categorical characteristics were compared using the Fisher’s exact test and numerical values with the unpaired *t* test for parametric variables, and the Mann–Whitney test for non-parametric variables. Normal distribution was checked with the Kolmogorov–Smirnov test. Two multivariable models (backward conditional models) were built, to examine the association of CRP at diagnosis with (i) characteristics at that time (first model) and with (ii) long-term outcomes, manifestations at any time during the disease course, and comorbidities (second model). The variables demonstrating statistical significance in the univariate analysis, as well as age and gender, were used as independent variables. It is important to stress that follow-up time was included in the second model. We acknowledge that the design of the second model does not conform to temporal sequencing. This model was constructed to explore associations between CRP at diagnosis and characteristics across the disease course, rather than causal relationships, and its results should be interpreted as such. Statistical significance for both the univariate and multivariable analyses was set at a *P*-value of <0.05. In the multivariable analyses, the results were expressed as odds ratios (ORs) along with 95% CIs. GraphPad Prism 5.00 (GraphPad Software, Inc., Boston, MA, USA) and SPSS 24.0 (SPSS software, Chicago, IL, USA) were used for the statistical analysis.

## Results

### Patient characteristics

Of the 923 initially included patients, 609 had documented CRP levels at diagnosis. The mean ± s.d. age was 55.7 ± 12.6 years, 55.0% were females, and the mean ± s.d. follow-up time was 8.3 ± 7.2 years. Among the patients included, 132 (21.7%) displayed normal CRP levels at diagnosis and 477 (78.3%) displayed elevated CRP levels. It is of note that 80.3% of the patients in the ‘normal’ CRP group still demonstrated normal CRP at the time of assessment.

### Comparison between patients with normal and elevated CRP—univariate analyses

#### Demographics and disease characteristics at PsA diagnosis

Regarding the demographic characteristics (including age, gender and smoking), there were no differences between the two groups, apart from the follow-up time (80.9 ± 76.7 *vs* 105.2 ± 87.6 months, *P* = 0.004) and the percentage of patients with a BMI of >25 kg/m^2^ (65.1% *vs* 77.1%, *P* = 0.014), which were significantly longer and higher, respectively, in the ‘elevated’ CRP group (Supplementary Table S1). Along the same lines, among the disease characteristics at the time of diagnosis, only the frequency of enthesitis differed, being higher in the ‘elevated’ CRP group (22.0% *vs* 35.6%, *P* = 0.004) (Supplementary Table S1).

#### Disease characteristics throughout the disease course and long-term outcomes

Throughout the disease course, significantly more patients in the ‘elevated’ CRP group compared with the ‘normal’ group displayed enthesitis (55.1% *vs* 44.9%, *P* = 0.045), hypertension (48.7% *vs* 35.5%, *P* = 0.011), hyperuricaemia (21.3% *vs* 8.3%, *P* < 0.001) (Supplementary Table S2) and new bone formation (but not erosions) as seen in hand x-rays (60.1% *vs* 31.2%, *P* < 0.0001) (Table 1). The mean time between PsA diagnosis and the conducting of hand x-rays was 3.27 ± 5.32 years in the ‘elevated’ group and 1.79 ± 2.89 in the ‘normal’ group, with no statistically significant difference being found between the groups (*P* = 0.16).

**Table 1:** Comparison of long-term outcomes in patients included in this study.

**Long term outcomes** ^a^	‘Normal’ CRP at Dx (21.7%) (*n* = 132)	‘Elevated’ CRP at Dx (78.3%) (*n* = 477)	*P*-value
Hand x-rays			
Erosion	28/78 (35.9%)	114/343 (33.2%)	0.691
New bone formation	24/77 (31.2%)	206/343 (60.1%)	0.0001
Spine and SI joint x-rays			
At least one positive XR^b^	25/91 (40%)	81/335 (28.4%)	0.585
Sacroiliitis^c^	9/80 (38.8%)	45/307 (28.3%)	0.587
Presence of syndesmophytes on spine	18/50 (36.0%)	51/220 (23.2%)	0.073
Arthroplasty	7/132 (5.3%)	11/477 (2.3%)	0.083
**Disease status at the time of assessment**			
Not in MDA	26/50 (52.0%)	87/235 (37.0%)	0.057
Active EMM^d^	64/132 (48.5%)	285/477 (59.7%)	0.022
Inflammatory activity^e^	33/132 (25.0%)	242/477 (50.7%)	0.0001
Persistent disease^f^	79/132 (59.8%)	382/477 (80.1%)	0.0001
D2M	25/132 (18.9%)	106/477 (22.2%)	0.473
TR	4/132 (3.0%)	26/477 (5.5%)	0.363
HAQ	0.357 (0.610), *n* = 73	0.276 (0.383), *n* = 359	0.156

Numbers are *N* (%). ^a^Based on the data available and collected at the time of assessment. ^b^At least one finding on x-rays consistent with sacroiliac involvement or spine involvement, as defined by syndesmophyte presence. ^c^≥Grade 2. ^dH^aving at least one active extra-musculoskeletal manifestation. ^e^CRP > 0.5 mg/dl and/or positive findings in MRI (SI joints and spine wherever) in the 6 months prior to time of assessment. ^f^(a) not being in MDA and/or (b) having at least one active extra-musculoskeletal manifestation, and/or (c) demonstrating ‘inflammatory activity’. D2M: difficult to manage; Dx: diagnosis; EMMs: extra-musculoskeletal manifestations; MDA: Minimal Disease Activity; TR: treatment refractory; XR: X-ray.

At the time of assessment, MDA was achieved at comparable percentages in the two groups, whereas ‘inflammatory activity’ (50.7% *vs* 25.0%, *P* = 0.0001) and at least one active extra-musculoskeletal manifestation (59.7% *vs* 48.5%, *P* = 0.022) were more common in the ‘elevated’ CRP group (Table 1).

Overall, evidence of ‘persistent disease’ was documented with a higher frequency in the ‘elevated’ CRP group (80.1% *vs* 59.8%, *P* < 0.001), while D2M and TR status were not significantly different (Table 1). Treatment received at the time of the assessment, as well as median number of b/tsDMARDs failed was similar between the ‘elevated’ and ‘normal’ CRP groups (Supplementary Table S3).

#### Comparison between patients with normal and ‘elevated’ CRP—multivariable analyses

Two multivariable analyses were performed, both with CRP status at diagnosis as the dependent variable. In the first model (Table 2), BMI > 25 and enthesitis at the time of diagnosis retained their association with ‘elevated’ CRP [OR 1.69 (CI = 1.05–2.72) and 2.04 (CI = 1.24–3.38), respectively], after adjustment for age and gender. In the second model (Table 2), hypertension (HTN) (OR 2.07, CI = 1.04–4.11), new bone formation in hand x-rays (OR 2.57, CI = 1.33–4.94), and ‘persistent disease’ (OR 4.36, CI = 2.22–8.59), were found to be associated with ‘elevated’ CRP (Table 2).

**Table 2:** Multivariable analyses. Two different models that have the CRP at the time of diagnosis as a dependent variable.

Characteristics at the time of diagnosis (Model 1)			
	*P*-value	OR	CI
BMI > 25 kg/m^2^	0.030	1.689	1.051–2.715
Enthesitis	0.005	2.044	1.237–3.375
**Long term effects and characteristics ever displayed (Model 2)**			
HTN	0.037	2.070	1.044–4.105
Hyperuricaemia	0.077	2.778	0.894–8.632
New bone formation^a^	0.005	2.565	1.331–4.942
Persistent disease^b^	<0.001	4.364	2.216–8.594

Variables entered on step 1 for characteristics at Dx: age, gender, BMI and enthesitis at Dx—variables entered on step 1 for long-term effects and characteristics ever displayed: age, gender, follow-up time in months, BMI, enthesitis ever displayed, HTN, hyperuricaemia, new bone formation, and persistent disease. ^a^As seen in hand x-rays. ^b^(a) not being in MDA and/or (b) having at least one active extra-musculoskeletal manifestation and/or (c) demonstrating ‘inflammatory activity’ (CRP > 0.5 mg/dl and/or positive findings in MRI (SI joints and spine wherever) in the 6 months prior to time of assessment). Dx: diagnosis; HTN: hypertension; MDA: Minimal Disease Activity; OR: odds ratio.

## Discussion

This study aimed to assess the potential association of CRP at the time of PsA diagnosis with long-term outcomes. We categorized the patients into two groups according to their CRP levels at diagnosis, ‘normal’ and ‘elevated’, in order to more efficiently explore potential associations that could be useful in clinical practice. In this large, real-life, contemporary patient cohort, elevated CRP at diagnosis was linked with a higher frequency of being overweight (BMI > 25) and having enthesitis at diagnosis, as well as with the development of HTN, new bone formation, and ‘persistent disease’ during follow-up.

In our study, 21.7% of the enrolled patients displayed normal CRP at PsA diagnosis and 78.3% displayed elevated CRP. This stated prevalence of ∼20% of the enrolled patients having a normal CRP at diagnosis can be explained by the low cut-off value (CRP ≤ 0.5 mg/dl), as well as the heterogeneity of the study population and the diverse clinical settings. However, considering the different cut-offs used in the literature, this is in agreement with the findings of other investigators. In a study by Haroon *et al.* [11], 56.5% of 283 patients enrolled had elevated CRP at their first rheumatology visit, with the cut-off set at CRP > 10.0 mg/l. In a subanalysis of the ADEPT randomized controlled trial [19], the baseline CRP was <1.0 mg/dl in 60% of the 152 patients in the placebo group and in 66% of the 144 patients in the adalimumab group, whereas in a study by van der Heijde *et al.* [18], with the cut-off for elevated CRP set at 2.87 mg/l, normal baseline CRP was observed in 36.4% of the patients in the ‘tofacitinib 5 mg twice daily’ group, 36.5% in the ‘tofacitinib 10 mg twice daily’ and 39.6% in the ‘adalimumab’ group. Therefore, depending on the cut-off used, approximately half of PsA patients do not have increased CRP at the time of diagnosis.

In our study, patients with elevated CRP at PsA diagnosis displayed a higher incidence of BMI > 25kg/m^2^. Obesity, an established PsA comorbidity, has been previously associated with elevated CRP levels in patients with PsA [11, 24]. It is considered that it mediates a low-grade inflammatory state due to the established endocrine and inflammatory properties of adipose tissue, which, among other tissues, secretes cytokines such as IL-6 and TNFα, subsequently leading to the elevation of inflammatory markers, such as CRP [24, 25]. Similarly, elevated CRP levels have previously been associated with HTN, another frequently documented comorbidity of PsA [26, 27]. Indeed, inflammation has been proven to have a crucial role in HTN development, with elevated CRP levels constituting a strong predictor for elevated blood pressure and normal CRP levels associated with hypertension remission irrespective of treatment, baseline systolic blood pressure, and anthropometric improvements [26, 28]. Given the independent associations of elevated CRP with BMI and HTN, a compelling link with the metabolic syndrome (MetS) emerges [25, 29]. MetS is a cluster of risk factors for cardiovascular disease, stroke and diabetes mellitus type 2, which include insulin resistance, central obesity, HTN, and atherogenic dyslipidaemia, and it has been shown to be highly prevalent among PsA patients [30, 31]. The components of the syndrome, particularly obesity and insulin resistance, mediate a proinflammatory state via the secretion of cytokines such as IL-6 and TNFα, which is proven to be detrimental in the syndrome’s pathophysiology [25]. Therefore, both in the general population as well as in PsA patients, the MetS has been associated with inflammation, as seen by elevated levels of CRP [31, 32].

In our study, we demonstrated for the first time that an elevated CRP at diagnosis is associated with new bone formation of the small joints of the hands, an outcome observed in approximately half of our patients. Regarding the impact of CRP in radiographic damage, in a study by Haroon *et al.* [11] that examined the association of CRP at the first rheumatology visit with disease outcomes, PsA patients with elevated baseline CRP displayed worse long-term radiographic damage (peripheral joint erosions and sacroiliitis on radiographs), compared with those with normal CRP [11]. Similarly, a subanalysis of the ADEPT trial concluded that baseline CRP is an independent predictor of radiographic progression (defined as a change in modified total Sharp score ΔmTSS > 0.5) in PsA, especially in untreated patients [19]. CRP was also associated with greater radiographic progression by van der Heijde *et al.*, who defined radiographic non-progression as a ≤5 increase from baseline modified total Sharp Score [18]. In our study, no association was found between CRP levels at diagnosis and radiology findings in the axis of the PsA patients enrolled.

We also found that, at diagnosis, elevated CRP appeared to be associated with the existence of enthesitis. Enthesitis is a common feature of PsA [33, 34] and is recognized as one of the key drivers of D2M/TR disease [35], although its pathogenetic mechanism in the disease is not entirely understood [33]. PG E2 initiates the inflammatory process, possibly inducing an acute stress response and activating the IL-13–IL-23 pathway [33, 34]. IL-17, along with TNFα—both produced due to IL-23 signalling—are key amplifiers of entheseal inflammation, with IL-17 causing further production of inflammatory mediators, such as IL-6 [33, 34], but also CRP itself [36]. Therefore, this link between IL-17 and CRP production may underlie the association between enthesitis and elevated CRP in our study [33]. However, in a previous study, CRP levels were not associated with enthesitis in PsA patients, nor with the extent of entheseal disease [37].

The value of CRP has been debated in the field of PsA for a long time. A significant percentage of PsA patients with clinically active disease do not show elevated CRP levels, raising questions regarding the utility of this acute-phase reactant in disease monitoring [17]. Indeed, when comparing DAPSA and cDAPSA in disease monitoring, Gonçalves *et al.* [16], Proft *et al.* [13] and van Mens *et al.* [15] documented a near-perfect agreement between the two indices in disease activity categorization, with the last also noting that the proportion of patients in remission was similar between those who had increased serum CRP levels and those who did not. It has been hypothesized that the frequent observation of normal CRP levels in PsA patients could be attributed to the minimal role of IL-6 in PsA pathogenesis, as it is one of the most significant cytokines in CRP signalling [37]. Sokolova *et al.* [37] and Gialouri *et al.* [17] suggest that alternative serum markers that detect different inflammatory pathways and different sites of inflammation could be indicative of existent inflammation when CRP is not. The aforementioned points, coupled with the fact that in our study ∼80% of the patients in the ‘normal’ CRP group maintained normal levels at the time of assessment, support the possibility of distinct immunologic phenotypes within PsA, with ‘elevated’ CRP patients representing a potentially separate PsA subgroup.

We acknowledge that this study has certain limitations. First, the cross-sectional design precludes causal inference, as does the model used for the multivariable analysis. Additionally, recall bias, missing data, and possibly inadequately controlled confounding variables are unavoidable due to the retrospective nature of the study. Nonetheless, in our study, <10% of the data was missing and all data was recorded in a predefined form with specific instructions given to the investigators. Patients with missing data were not included in the multivariable analyses. Selection bias is also a possibility, particularly in radiographic assessments, which were not performed systematically for all patients and were likely performed based on clinical indications. Also, no central reading of the x-rays was conducted, which may have led to a bias. Moreover, the findings for this study were collected in a specific electronic form that the investigators were asked to fill in. Another limitation is that, even though psoriasis was present in all patients included in the study, data on disease severity [Psoriasis Area and Severity Index (PASI) or BSA] were not sufficiently complete to be included in our analysis. Furthermore, we acknowledge that the ‘persistent disease’ definition includes CRP levels at the time of assessment, introducing potential overlap with CRP at diagnosis. However, the potential for circularity is limited, as CRP levels represent one component of a composite definition that includes multiple other variables. In addition, CRP in the ‘persistent disease’ definition refers to a measurement within the last 6 months prior to the time of assessment, and given that the follow-up time was shown to be 8.3 ± 7.2 years, it was not necessarily related to CRP at diagnosis. Finally, the longer follow-up time in the ‘elevated CRP’ group could increase the incidence of adverse outcomes. To partially account for this, it was included in the multivariable analysis. Also, we note that the mean time between PsA diagnosis and the conducting of hand x-rays showed no statistically significant difference between the two groups.

This study also has multiple strengths. A major strength of this study is that it reflects a real-world cohort of patients with PsA, including individuals with diverse disease characteristics. The sample analysed was considerably large and derived from a multicentre registry. Furthermore, to our knowledge, this is the first study that has included EULAR’s definition of D2M and TR PsA. Importantly, the analysed population was divided by CRP levels at diagnosis, in treatment-naïve patients; hence, the potential confounding effect of immunomodulatory treatment is not present in the CRP measurements, allowing for a clearer assessment of the marker’s prognostic value.

In conclusion, CRP levels at PsA diagnosis appear to have clinical significance, being associated with adverse outcomes, such as new bone formation and ‘persistent disease’. The levels of this acute-phase reactant at diagnosis could serve as an identifier of separate PsA subgroups with potentially different disease trajectories. Thus, given the clinical heterogeneity of PsA and the challenges it poses for treatment selection across multiple disease domains [7], this biomarker may contribute to improved risk stratification and a more personalized treatment approach, ultimately optimizing patient care. In this context, further research is warranted to define the prognostic utility of CRP in PsA and to determine its value as a biomarker for disease burden, treatment response, and long-term clinical outcomes.

Institutional Review Board approval was provided by the Joint Rheumatology Program (‘Laiko Hospital’ No.: 780/2021) and by the local institutional boards of participating centers. All patients provided written informed consent.

## Supplementary Material

keag398_Supplementary_Data

## Data Availability

The Greek Rheumatology Society has the ownership of the data. These are available upon reasonable request to the corresponding author.

## References

[1] Azuaga AB , Ramírez J, Cañete JD. Psoriatic arthritis: pathogenesis and targeted therapies. Int J Mol Sci 2023;24. doi: 10.3390/ijms24054901 PMC10003101 36902329

[2] Coates LC , Helliwell PS. Psoriatic arthritis: state of the art review. Clin Med 2017;17:65–70. doi: 10.7861/clinmedicine.17-1-65 PMC6297592 28148584

[3] Gialouri CG , Evangelatos G, Zhao SS et al Depression and anxiety in a real-world psoriatic arthritis longitudinal study: should we focus more on patients’ perception? Mood disorders in psoriatic arthritis/ C.G. Gialouri et al. Clin Exp Rheumatol 2023;41:159–65. 10.55563/clinexprheumatol/8qxo80 35819812

[4] Panagiotopoulos A , Fragoulis GE. Comorbidities in psoriatic arthritis: a narrative review. Clin Ther 2023;45:177–89. doi: 10.1016/j.clinthera.2023.01.006 36737317

[5] Ogdie A , Weiss P. The epidemiology of psoriatic arthritis. Rheum Dis Clin North Am 2015;41:545–68. doi: 10.1016/j.rdc.2015.07.001 PMC4610151 26476218

[6] Fragoulis GE , Siebert S. The role of IL-23 and the use of IL-23 inhibitors in psoriatic arthritis. Musculoskeletal Care 2022;20 Suppl 1:S12–S21. doi: 10.1002/msc.1694 PMC9825973 36069174

[7] Spanopoulou C , Katsimbri P. Psoriatic Arthritis: developments in 2025. Mediterr J Rheumatol 2026;37:12–20. doi: 10.31138/mjr.200925.per PMC12835932 41609290

[8] Rizo-Téllez SA , Sekheri M, Filep JG. C-reactive protein: a target for therapy to reduce inflammation. Front Immunol 2023;14:1237729. doi: 10.3389/fimmu.2023.1237729 PMC10410079 37564640

[9] Bray C , Bell LN, Liang H et al Erythrocyte sedimentation rate and c-reactive protein measurements and their relevance in clinical medicine. WMJ 2016;115:317–21. 29094869

[10] Litao MKS , Kamat D. Erythrocyte sedimentation rate and c-reactive protein: how best to use them in clinical practice. Pediatr Ann 2014;43:417–20. doi: 10.3928/00904481-20140924-10 25290132

[11] Haroon M , Gallaghar P, Ahmad M, FitzGerald O. Elevated CRP even at the first visit to a rheumatologist is associated with long-term poor outcomes in patients with psoriatic arthritis. Clin Rheumatol 2020;39:2951–61. doi: 10.1007/s10067-020-05065-9 32242283

[12] Gialouri CG , Fragoulis GE. Disease activity indices in psoriatic arthritis: current and evolving concepts. Clin Rheumatol 2021;40:4427–35. doi: 10.1007/s10067-021-05774-9 34003419

[13] Proft F , Schally J, Brandt HC et al Evaluation of the Disease Activity index for PSoriatic Arthritis (DAPSA) with a quick quantitative C reactive protein assay (Q-DAPSA) in patients with psoriatic arthritis: a prospective multicentre cross-sectional study. RMD Open 2022;8:4–5. doi: 10.1136/rmdopen-2022-002626 PMC9639152 36323487

[14] Coates LC , Gottlieb AB, Merola JF et al Comparison of different remission and low disease definitions in psoriatic arthritis and evaluation of their prognostic value. J Rheumatol 2019;46:160–5. doi: 10.3899/jrheum.180249 30323006

[15] van Mens LJJ , van de Sande MGH, van Kuijk AWR, Baeten D, Coates LC. Ideal target for psoriatic arthritis? Comparison of remission and low disease activity states in a real-life cohort. Ann Rheum Dis 2018;77:251–7. doi: 10.1136/annrheumdis-2017-211998 29080861

[16] Gonçalves RSG , de Almeida Martins LM, de Ataide Mariz H, Dantas AT, Duarte ALBP. DAPSA versus cDAPSA: do we need to use CRP? Ann Rheum Dis 2020;79:e142. doi: 10.1136/annrheumdis-2019-215960 31331922

[17] Gialouri CG , Evangelatos G, Pappa M et al Normal C-reactive protein in active psoriatic arthritis: results from real-world clinical practice. Ther Adv Musculoskelet Dis 2022;14:1759720X221122417. doi: 10.1177/1759720X221122417 PMC9445451 36081746

[18] van der Heijde D , Gladman DD, FitzGerald O et al Radiographic progression according to baseline C-reactive protein levels and other risk factors in psoriatic arthritis treated with tofacitinib or adalimumab. J Rheumatol 2019;46:1089–96. doi: 10.3899/jrheum.180971 30824647

[19] Gladman DD , Mease PJ, Choy EHS et al Risk factors for radiographic progression in psoriatic arthritis: subanalysis of the randomized controlled trial ADEPT. Arthritis Res Ther 2010;12:R113. doi: 10.1186/ar3049 PMC2911906 20537151

[20] Borst C , Alasti F, Smolen JS, Aletaha D. Role of clinical and biochemical inflammation in structural progression of patients with psoriatic arthritis. RMD Open 2021;7:2–4. doi: 10.1136/rmdopen-2021-002038 PMC8655607 34880129

[21] Punzi L , Punzi L, Podswiadek M, Oliviero F, Lonigro A, Modesti V et al Laboratory findings in psoriatic arthritis. 10.4081/reumatismo.2007.1s.52 17828345

[22] Fragoulis GE , Papagoras C, Gazi S et al Disease profile and achievement of therapeutic goals in a modern, nationwide cohort of 923 patients with psoriatic arthritis. Mediterr J Rheumatol 2023;34:418–26. doi: 10.31138/mjr.301223.dpa PMC10815515 38282940

[23] Marzo-Ortega H , Harrison SR, Fragoulis GE et al EULAR points to consider and consensus definitions for difficult-to-manage and treatment-refractory psoriatic arthritis. Ann Rheum Dis 2026;85:61–74. doi: 10.1016/j.ard.2025.10.002 41168056

[24] Kumthekar A, Ogdie A. Obesity and psoriatic arthritis: a narrative review. Rheumatol Ther 2020;7:447–56. doi: 10.1007/s40744-020-00215-6 PMC7410935 32495313

[25] Fahed G , Aoun L, Bou Zerdan M et al Metabolic Syndrome: updates on Pathophysiology and Management in 2021. Int J Mol Sci 2022;23. doi: 10.3390/ijms23020786 PMC8775991 35054972

[26] Carbone F , Elia E, Casula M et al Baseline hs-CRP predicts hypertension remission in metabolic syndrome. Eur J Clin Invest 2019;49:e13128. doi: 10.1111/eci.13128 31091356

[27] Gupta S , Syrimi Z, Hughes DM, Zhao SS. Comorbidities in psoriatic arthritis: a systematic review and meta-analysis. Rheumatol Int 2021;41:275–84. doi: 10.1007/s00296-020-04775-2 PMC7835184 33423070

[28] Kuppa A , Tripathi H, Al-Darraji A, Tarhuni WM, Abdel-Latif A. C-reactive protein levels and risk of cardiovascular diseases: a two-sample bidirectional mendelian randomization study. Int J Mol Sci 2023;24:14–15. doi: 10.3390/ijms24119129 PMC10252732 37298077

[29] Cho Y , Lee SY. Useful biomarkers of metabolic syndrome. Int J Environ Res Public Health 2022;19:2. doi: 10.3390/ijerph192215003 PMC9690835 36429722

[30] Mok CC , Ko GTC, Ho LY et al Prevalence of atherosclerotic risk factors and the metabolic syndrome in patients with chronic inflammatory arthritis. Arthritis Care Res (Hoboken) 2011;63:195–202. doi: 10.1002/acr.20363 20890981

[31] Urruticoechea-Arana A , Castañeda S, Otón T et al Prevalence of metabolic syndrome in psoriatic arthritis: systematic literature review and results from the CARMA cohort. J Clin Rheumatol 2022;28:e388–e396. doi: 10.1097/RHU.0000000000001738 35192593

[32] Papagoras C , Voulgari PV, Drosos AA. Cardiovascular disease in spondyloarthritides. Curr Vasc Pharmacol 2020;18:473–87. doi: 10.2174/1570161117666190426164306 31330576

[33] Schett G , Lories RJ, D’Agostino M-A et al Enthesitis: from pathophysiology to treatment. Nat Rev Rheumatol 2017;13:731–41. doi: 10.1038/nrrheum.2017.188 29158573

[34] Araujo EG , Schett G. Enthesitis in psoriatic arthritis (Part 1): pathophysiology. Rheumatology (Oxford) 2020;59:I10–i14. doi: 10.1093/rheumatology/keaa039 PMC7065460 32159793

[35] Harrison SR , Fragoulis GE, Michelena X et al Prevalence, outcomes, and predictive factors: a systematic literature review to inform the development of EULAR Points to Consider for the definition of Difficult-to-Manage and Treatment-Refractory psoriatic arthritis. EULAR Rheumatol Open 2026;2:100043. doi: 10.1016/j.ero.2025.07.007 PMC13425173 42540123

[36] Patel DN , King CA, Bailey SR et al Interleukin-17 Stimulates C-reactive Protein Expression in Hepatocytes and Smooth Muscle Cells via p38 MAPK and ERK1/2-dependent NF-κB and C/EBPβ Activation. J Biol Chem 2007;282:27229–38. doi: 10.1074/jbc.M703250200 PMC3818724 17652082

[37] Sokolova MV , Simon D, Nas K et al A set of serum markers detecting systemic inflammation in psoriatic skin, entheseal, and joint disease in the absence of C-reactive protein and its link to clinical disease manifestations. Arthritis Res Ther 2020;22:3–5. doi: 10.1186/s13075-020-2111-8 PMC7017480 32051028

