# Supplementary material for: CRP at diagnosis in psoriatic arthritis: what it means and associations with long-term outcomes
Source: Rheumatology (Oxford). 2026 Aug 7;65(9):keag398. doi: 10.1093/rheumatology/keag398 (PMC13546032; doi:10.1093/rheumatology/keag398)
Supplement: Supplementary file 1 [file keag398_supplementary_data.zip › rhe-26-0594-File003.docx]

| **Characteristic** | **“normal” CRP**  **at Dx (21.7%)**  **(n=132)** | **“elevated” CRP**  **at Dx (78.3%)**  **(n=477)** | **p-value** |
| --- | --- | --- | --- |
| **Demographics** | | | |
| Age, mean (SD), years | 55.0 (12.4) | 56.0 (12.6) | 0.503 |
| Gender (♀), n (%) | 81/132 (61.4%) | 254/477 (53.2%) | 0.114 |
| BMI, mean (SD), kg/m^2^ | 28.2 (6.3), n=109 | 29.0 (5.7), n=436 | 0.061 |
| BMI ≥ 25kg/m^2^, n (%) | 71/109 (65.1%) | 336/436 (77.1%) | 0.014 |
| Follow-up time, mean (SD) months | 80.9 (76.7), n=112 | 105.2 (87.6), n=421 | 0.004 |
| Smoking (now or ever), n (%) | 72/125 (57.6%) | 241/453 (53.2%) | 0.418 |
| **Characteristics at the time of PsA diagnosis** | | | |
| Age, mean (SD) years | 48.8 (14.0), n=112 | 47.3 (12.6), n=421 | 0.228 |
| Peripheral Arthritis, n (%) | 106/132 (80.3%) | 360/477 (75.5%) | 0.296 |
| Monoarthritis, n (%) | 11/132 (8.3%) | 20/477 (4.2%) | 0.071 |
| Oligoarthritis, n (%) | 41/132 (31.1%) | 118/477 (24.7%) | 0.147 |
| Polyarthritis, n (%) | 50/132 (37.9%) | 170/477 (35.6%) | 0.682 |
| Enthesitis, n (%) | 28/127 (22.0%) | 165/463 (35.6%) | 0.004 |
| Nail involvement, n (%) | 36/127 (28.3%) | 163/457 (35.7%) | 0.139 |
| Dactylitis, n (%) | 22/128 (17.2%) | 98/459 (21.4%) | 0.324 |
| Axial disease ֘, n (%) | 24/132 (18.2%) | 71/477 (14.9%) | 0.125 |
| IBD, n (%) | 1/132 (0.8%) | 3/477 (0.6%) | 1.000 |
| Uveitis, n (%) | 1/127 (0.8%) | 5/449 (1.1%) | 1.000 |

**Supplementary Table S1:** Demographics and disease characteristics at the time of PsA diagnosis. Comparison between “CRP-high” and “CRP-low” patients. CRP: C-Reactive Protein, BMI: Body Mass Index, Dx: diagnosis, SD: Standard Deviation, IBD: Inflammatory Bowel Disease of patients included in this study.

֘axial disease was defined as symptoms of inflammatory back pain accompanied by radiologic findings in x-rays or MRIs

|  | **“normal” CRP**  **at Dx (21.7%)**  **(n=132)** | **“elevated” CRP**  **at Dx (78.3%)**  **(n=477)** | **p-value** |
| --- | --- | --- | --- |
| **Characteristics ever-present throughout disease course** | | | |
| Enthesitis, n (%) | 57/127 (44.9%) | 255/463 (55.1%) | 0.045 |
| Dactylitis, n (%) | 35/128 (27.3%) | 168/459 (36.6%) | 0.058 |
| Nail involvement, n (%) | 50/127 (39.4%) | 209/457 (45.7%) | 0.226 |
| Axial disease ֘, n (%) | 43/132 (32.6%) | 122/477 (25.6%) | 0.121 |
| DM, n (%) | 20/120 (16.7%) | 75/454 (16.5%) | 1.000 |
| Coronary Vascular Disease, n (%) | 6/121 (5.0%) | 23/448 (5.1%) | 1.000 |
| Hypercholesterolemia, n (%) | 50/119 (42.0%) | 193/439 (44.0%) | 0.754 |
| Dyslipidaemia, n (%) | 53/120 (44.2%) | 207/457 (45.3%) | 0.837 |
| Hypertension, n (%) | 44/124 (35.5%) | 221/454 (48.7%) | 0.011 |
| Depression, n (%) | 31/121 (25.6%) | 100/446 (22.4%) | 0.467 |
| IBD, n (%) | 3/128 (2.3%) | 9/456 (2.0%) | 0.731 |
| Uveitis, n (%) | 4/127 (3.1%) | 14/449 (3.1%) | 1.000 |
| Hyperuricemia, n (%) | 10/121 (8.3%) | 95/446 (21.3%) | 0.001 |
| MACCE, n (%) | 8/132 (6.1%) | 41/477 (8.6%) | 0.469 |

**Supplementary Table S2:** Disease characteristics through-out disease course. CRP: C-Reactive Protein, Dx: diagnosis, DM: Diabetes Mellitus, IBD: Inflammatory Bowel Disease, MACCE: Major Adverse Cardiac and Cerebrovascular Events (including coronary artery disease, stroke, death related to CVD)

֘axial disease was defined as symptoms of inflammatory back pain accompanied by radiologic findings in x-rays or MRIs

|  | **“normal” CRP**  **at Dx (21.7%)**  **(n=132)** | **“elevated” CRP**  **at Dx (78.3%)**  **(n=477)** |
| --- | --- | --- |
| **At the time of assessment** |  |  |
| cDMARDs | 68 (51.5%) | 281 (58.9%) |
| bDMARDs | 77 (58.3%) | 262 (54.9%) |
| tsDMARDs | 17 (12.9%) | 35 (7.4%) |
| Combination c+b | 38 (28.8%) | 134 (28.1%) |
| Combination c+ts | 8 (6.1%) | 17 (3.6%) |
| **Failed b/ts DMARDS** |  |  |
| bDMARDs, median (IQR) number of failed drugs | 0 (1) | 0 (1) |
| tsDMARDs, median (IQR) number of failed drugs | 0 (0) | 0 (0) |

**Supplementary Table S3 – Past and present anti-rheumatic treatments received by the patients studied**

Numbers are n (%)

CRP: C-Reactive Protein, Dx: Diagnosis, cDMARDs: conventional Disease-modifying antirheumatic drugs, bDMARDs: biologic Disease-modifying antirheumatic drugs, tsDMARDs: targeted synthetic Disease-modifying antirheumatic drugs, IQR: Interquartile Range
